# Characterization of macrophages in head and neck squamous cell carcinoma and development of MRG-based risk signature

**DOI:** 10.1038/s41598-024-60516-6

**Published:** 2024-04-30

**Authors:** Lei Liu, Qiang Liu

**Affiliations:** Department of Otorhinolaryngology, The Affiliated Traditional Chinese Medicine Hospital, Southwest Medical University, Luzhou, 646000 China

**Keywords:** Head and neck squamous cell carcinoma, Macrophages, Tumor-associated macrophages, Prognostic model, Immunotherapy, Cancer, Computational biology and bioinformatics

## Abstract

Macrophages are immune cells in the TME that can not only inhibit angiogenesis, extracellular matrix remodeling, cancer cell proliferation, and metastasis but also mediate the phagocytosis and killing of cancer cells after activation, making them key targets in anti-tumor immunotherapy. However, there is little research on macrophages and their relation to disease prognosis in HNSCC. Initially, we collected scRNA-seq, bulk RNA-seq, and clinical data. Subsequently, we identified macrophages and distinguished MRGs. Using the K-means algorithm, we performed consensus unsupervised clustering. Next, we used ssGSEA analysis to assess immune cell infiltration in MRG clusters. A risk model was established using multivariate Cox analysis. Then, Kaplan–Meier, ROC curves, univariate and multivariate COX analyses, and C-index was used to validate the predictive power of the signature. The TIDE method was applied to assess the response to immunotherapy in patients diagnosed with HNSCC. In addition, drug susceptibility predictions were made for the GDSC database using the calcPhenotype function. We found that 8 MRGs had prognostic potential. Patients in the MRG group A had a higher probability of survival, and MRG clusters A and B had different characteristics. Cluster A had a higher degree of expression and infiltration in MRG, indicating a closer relationship with MRG. The accuracy of the signature was validated using univariate and multivariate Cox analysis, C-index, and nomogram. Immune landscape analysis found that various immune functions were highly expressed in the low-risk group, indicating an improved response to immunotherapy. Finally, drugs with high sensitivity to HNSCC (such as 5-Fluorouracil, Temozolomide, Carmustine, and EPZ5676) were explored and analyze the malignant characteristics of HNSCC. We constructed a prognostic model using multivariate Cox analysis, consisting of 8 MRGs (TGM2, STC1, SH2D3C, PIK3R3, MAP3K8, ITGA5, ARHGAP4, and AQP1). Patients in the low-risk group may have a higher response to immunotherapy. The more prominent drugs for drug selection are 5-fluorouracil, temozolomide and so on. Malignant features associated with HNSCC include angiogenesis, EMT, and the cell cycle. This study has opened up new prospects for the prognosis, prediction, and clinical treatment strategy of HNSCC.

## Introduction

Head and neck squamous cell carcinoma (HNSCC) is the main type (more than 90%) of head and neck tumors (common sites: mouth, pharynx, tongue, larynx, etc.)^1–3^. The major risk factors for the disease are smoking, alcohol abuse, local repeated irritation, viral infection, and so on^3–6^. Traditional surgery, radiotherapy, and chemotherapy are still important comprehensive treatment options for HNSCC, but with the fast progress of targeted therapy and immunotherapy study, the comprehensive treatment of this disease (especially advanced HNSCC) has gradually approached immunotherapy^7–9^. In previous studies, targeted immunotherapy increased survival in patients with HNSCC, but fewer than one in five patients had a long-lasting treatment response^10–13^. Given this situation, we believe that investigating new immunotherapies by studying key players in the tumor microenvironment (TME) of HNSCC can further improve the immune response of tumor cells and, accordingly, the therapeutic efficacy of HNSCC.

Macrophages are derived from monocytes, which are a type of white blood cell. As the name implies, tumor-associated macrophages (TAMs) are immune cells present in the TME. They can inhibit angiogenesis, extracellular matrix remodeling, cancer cell proliferation, and metastasis. Additionally, they can mediate the phagocytosis and killing of cancer cells after activation, so they are key targets in anti-tumor immunotherapy^14^. Macrophage-related genes (MRGs) can determine the morphology and function of macrophages, and MRGs can exert an essential function in tumor recognition, phagocytosis, and cytokine release by regulating macrophages, which are important in controlling and eliminating tumors^15,16^. Exosomes can activate the NF-κB pathway in macrophages, thereby enhancing the expression of pro-inflammatory elements, thereby promoting the proliferation and metastasis of gastric cancer cells^17^. The risk scoring system formed by using 11 MRGs predicts the prognosis of lung cancer patients and evaluates their immune response. State of infiltration^18^. In colon cancer, four central genes were recognized as associated with M2 macrophages, and the M2I score was established accordingly, among which TMB, MSI, and sensitivity are higher^19^. There are countless similar studies that have demonstrated that macrophages, which are regulated by macrophage-related genes, can play an important in tumor regulation and prognosis.

The literature has pointed out that in HNSCC, TAM is related to poor prognosis because it promotes tumor progression and suppresses immune response through innate and adaptive immune mechanisms, but TAM as a double-edged sword can also inhibit tumor cells by depleting immunosuppressive function or stimulating anti-tumor ability^20^. In this study, the cell data of HNSCC will be obtained through public datasets, and then the data will be analyzed to obtain "MRGs that can judge prognosis" and "disease prognosis prediction models based on MRGs", so as to provide more scientific medical suggestions for the intervention of HNSCC patients.

## Methods

### Preparation of data

**Figure 1:**
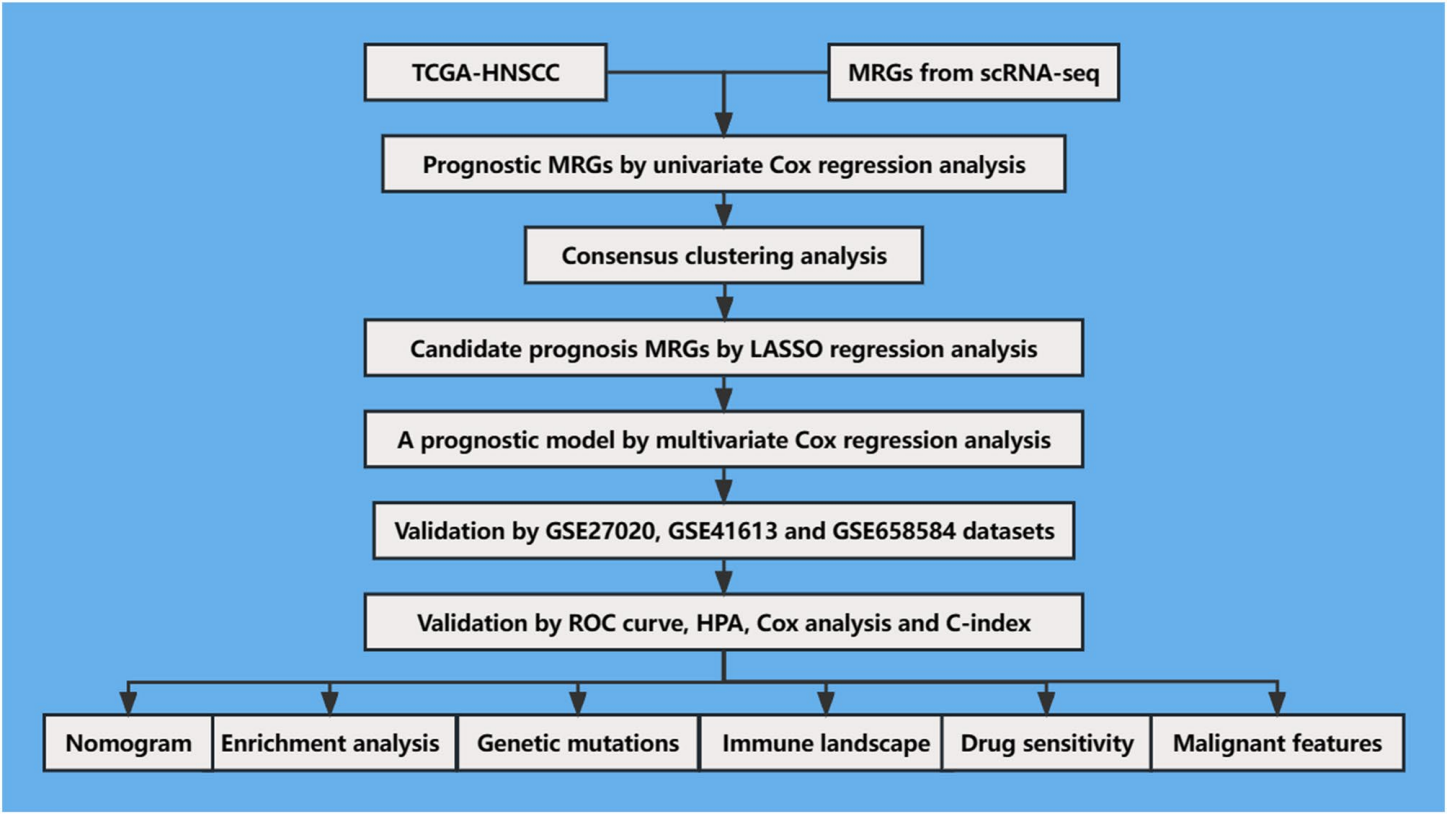
The overall workflow of the study.

This study utilized multiple publicly available datasets to investigate the molecular mechanisms underlying HNSCC. Specifically, scRNA-seq data from 18 HNSCC samples were obtained from the GEO-GSE103322 dataset, resulting in a total of 5902 single cells being analyzed^21^. RNA-seq and clinical data for HNSCC were also obtained from the TCGA-HNSCC, GSE27020, GSE41613, and GSE65858 datasets. After removing incomplete and duplicate data, the study analyzed 511 HNSCC samples from TCGA-HNSCC, 109 from GEO-GSE27020, 97 from GEO-GSE41613, and 270 from GEO-GSE65858^22–25^. Additionally, RNA-seq and clinical data were obtained from 8739 samples representing 32 different types of tumors from USCS Xena^22–25^. Figure 1 illustrates the general workflow of this research.

### Manipulation of scRNA-seq data

Firstly, we implemented the "Seurat" package to create a Seurat object, which served as the foundation for analyzing the scRNA-seq data^27^. We conducted quality control procedures based on thresholds established in previous studies^21^. Subsequently, we processed cell cycle effects, performed data normalization, employed dimensionality reduction techniques (1:30), proceeded with clustering analysis (resolution = 0.5), and allocated cell annotations^28,29^. To recognize MRGs, we employed the "FindAllMarkers" function, which allowed us to recognize highly variable genes meeting specific criteria (logFC ≥ 0.3, min.pct = 0.3, and diff.pct ≥ 0.2) within the macrophage subset. These genes were deemed as MRGs, denoting their potential involvement in macrophage function and biology. The utilization of the Seurat package, in combination with established guidelines and statistical approaches, ensures the robustness and validity of the results.

### Recognition of prognostic MRGs

To investigate the prognostic significance of MRGs in HNSCC, we performed univariate Cox analysis (*p* < 0.05), and aimed at identifying MRGs significantly associated with survival outcomes in HNSCC. After this step, we employed a comprehensive analytical strategy that involved an in-depth examination of the inter-expression relationship between the CNV landscape and the recognized prognostic MRGs^30,31^.

### Consensus clustering analysis

To elucidate the clinically and pathologically relevant aspects of the recognized MRGs, we utilized the K-means algorithm with consensus unsupervised clustering analysis through the R package "ConsensusClusterPlus"^32^. This method allowed us to identify different clusters of samples according to the expression levels of the MRGs, with the selection of optimal K values^33^. We evaluated differences in survival outcomes among the MRG clusters with survival analyses, enabling us to determine the potential clinical significance of these MRG clusters. To explore the distribution of MRG clusters, we employed dimensionality reduction techniques, including PCA, tSNE, and UMAP analyses. Besides, we evaluated the relationship between MRG clusters and specific clinical features. In summary, these analytical approaches enabled us to gain a comprehensive understanding of the potential significance of the recognized MRGs, with the potential to inform tailored therapeutic interventions and improve clinical outcomes for HNSCC patients.

### Exploration of immune status and enrichment analysis

Firstly, we conducted an analysis of the expression patterns of MRGs among MRG clusters, aiming to recognize distinct regulatory patterns among MRGs. By examining the expression patterns, we intended to obtain a comprehensive understanding of the different regulatory mechanisms at play in HNSCC. Additionally, we used ssGSEA to assess the levels of immune cell infiltration in MRG clusters^34^. We sought to discern any significant differences in immune cell infiltration between the clusters with the Wilcoxon test. This method would provide valuable information regarding the potential influence of prognostic MRGs on the TME and immune response in HNSCC. Furthermore, we implemented the GSVA method to assess the activation status of KEGG pathways^34^. By employing this algorithm, we aimed to identify the key pathways related to each cluster. To visualize the results and highlight the most significant enrichment outcomes, we generated a heat map displaying the top 30 enrichment results.

### Construction of prognostic MRG model

We implemented LASSO regression analysis to screen the candidate MRGs and diminish any irrelevant and redundant factors. By introducing a penalty term to the conventional regression model, the LASSO analysis allowed us to reduce the complexity of the signature by shrinking the coefficients of unimportant genes to zero while keeping the most significant genes^35^. Furthermore, we applied multivariate Cox analysis to develop a prognostic model^36^.

### Validation of prognostic MRG model

Survival analysis was applied to examine the differences in survival. Furthermore, the accuracy of the prognostic signature was assessed using ROC curves, which allowed for an evaluation of its predictive performance over time^37^. To ensure the generalizability of the model, HNSCC samples from multiple datasets, including GSE27020, GSE41613, and GSE65858, were utilized for external validation. The validity of the model was further examined using the HPA database, which provided information on the protein expression levels of the identified prognostic MRGs in both normal and HNSCC tissues. This analysis offered worthwhile insights into the potential utility of the model in guiding treatment decisions for specific patient populations. Moreover, both univariate and multivariate Cox analyses were conducted to assess the independent predictive power of the prognostic model. The C-index was employed to quantify the discriminatory power of the signature with respect to traditional clinical features. This assessment provided a quantitative measure of the model's predictive performance and contributed to the determination of its added value beyond established prognostic factors. Lastly, a nomogram, incorporating both the clinical data and the signature, was developed to predict the survival rates of HNSCC. By incorporating multiple prognostic factors into a single model, the nomogram provided a practical tool for individualized patient prognosis estimation.

### Enrichment and gene mutation analyses

This study employed advanced methodologies to identify DEGs across distinct risk groups. To ensure the robustness of the findings, DEGs were identified using stringent criteria, including a threshold of |logFC >  = 1.5| and a FDR of less than 0.05. Subsequently, the identified DEGs underwent GO and KEGG analyses (*p* < 0.05), to elucidate their biological functions and involve signaling pathways^38–41^. To further explore the genomic landscape of HNSCC and its association with different risk scores, the "Maftools" package was used^42^. This tool enabled the identification and comparison of the mutation burden between various risk score groups. By quantifying the number of mutations present, the implication of genomic alterations in contributing to different levels of risk in HNSCC could be investigated.

### Exploration of immunization status

We employed a range of analytical approaches to assess immune cell infiltration. Various methods were used to deconvolve and quantify the immune cell composition in the TME^43–49^. The ssGSEA approach was employed to investigate the immune function of the risk groups. This method allowed for the quantification of immune function scores, which reflect the overall immune activity and response within each risk group. Moreover, the expression levels of ICGs within the identified risk groups were examined. ICGs play a crucial role in regulating the immune response and can impact the success of immunotherapeutic interventions. The TIDE method was employed to forecast the immunotherapy response^50^. This algorithm takes into account various immune-related factors, including tumor immune evasion and cytotoxic immune infiltration, to forecast the likelihood of an immunotherapeutic response.

### Recognition of medicines

To investigate the efficacy of different drugs against HNSCC, we utilized the "calcPhenotype" function in the "oncoPredict" package to forecast drug sensitivity^51^. By applying the approach to the GDSC database, the study was able to recognize the efficacy of specific drugs for HNSCC.

### Correlation with malignant features

The z-score method is used to normalize gene expression data, facilitates comparative analyses between different samples, and enables calculation of the activity of specific pathways and integration of the expression of characteristic genes^26,52^. Based on the "GSVA" package, we explored the relationship between the MRG signature and angiogenesis, cell cycle regulation, and EMT by comparing them with the pearson algorithm^53,54^.

## Result

### Recognition of MRGs

**Figure 2:**
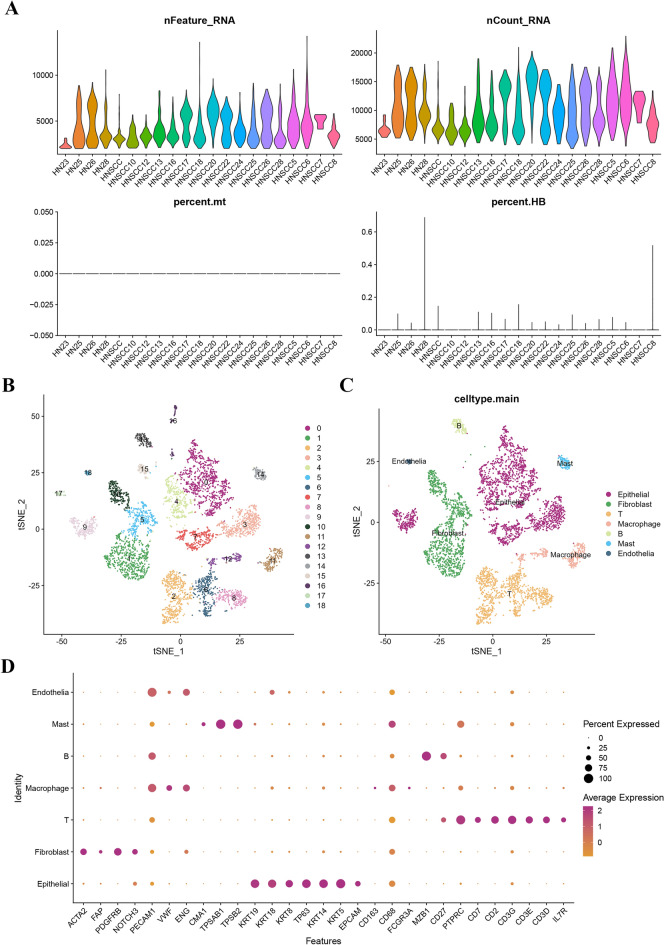
(**A**) 5,902 cells from the GSE103322 database was analyzed after quality control. (**B**) The distribution of different cell populations. (**C**) Cell annotations for each cluster. (**D**) The marker genes.

This study investigated the molecular characteristics of HNSCC using scRNA-seq analysis. 18 HNSCC samples were collected, and a dataset of 5902 cells from the GSE103322 database was analyzed after quality control (Fig. 2A). The scRNA-seq data were normalized, reduced dimensionality, and clustered using tSNE plot analysis, revealing the distribution of different cell populations (Fig. 2B). Cell annotations for each cluster were performed (Fig. 2C) and bubble plots were generated to demonstrate the expression of genes (Fig. 2D). Additionally, the "FindAllMarkers" function was utilized to recognize 216 significantly differentially expressed MRGs (Table S1).

### Recognition of prognostic MRGs

**Figure 3:**
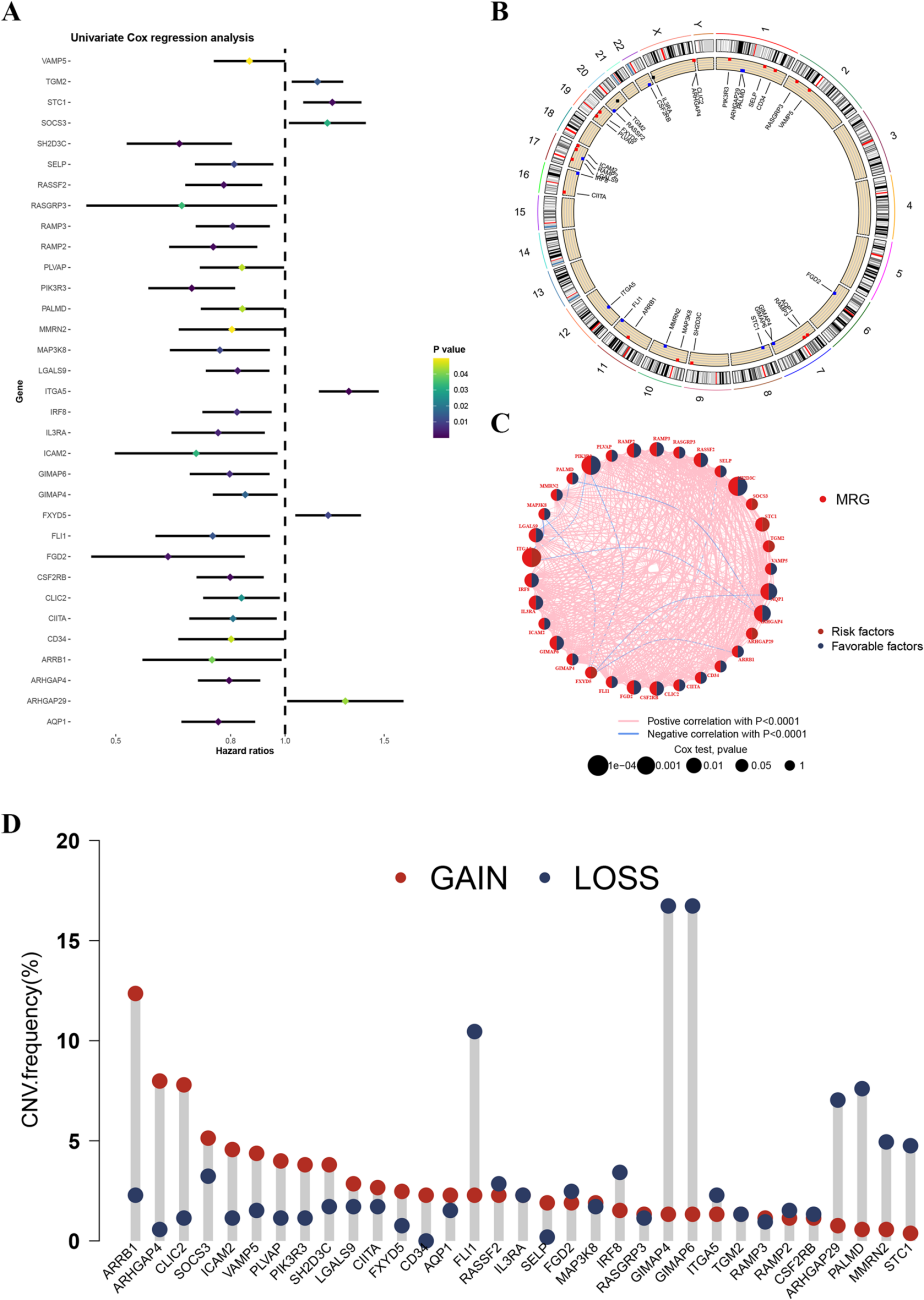
(**A**) 33 prognostic MRGs recognized by univariate Cox analysis. (**B**) The location of 33 MRGs on chromosomes. (**C**) Interaction among 33 MRGs. (**D**) The CNV frequency of 33 MRGs in HNSCC.

We conducted a univariate Cox analysis, showing that 33 MRGs were significantly related to overall survival (Fig. 3A). To understand the potential genetic alterations affecting these MRGs, the locations of CNVs on chromosomes were tagged (Fig. 3B). A network analysis was performed to explore the interconnections and regulatory relationships among the MRGs, providing a comprehensive understanding of their biological functions and potential interactions, as depicted in Fig. 3C. Furthermore, the CNVs of the 33 identified MRGs were investigated. Among these genes, ARRB1, ARHGAP4, CLIC2, SOCS3, ICAM2, VAMP5, PLVAP, PIK3R3, SH2D3C, LGALS9, CIITA, FXYD5, CD34, AQP1, SELP, MAP3K8, RASGRP3, and RAMP3 showed a higher frequency of amplification CNVs, indicating potential oncogenic roles for these genes. On the other hand, LI1, RASSF2, IL3RA, FGD2, IRF8, GIMAP4, GIMAP6, ITGA5, TGM2, RAMP2, CSF2RB, ARHGAP29, PALMD, MMRN2, and STC1 exhibited a higher frequency of missing CNVs for deletions, suggesting potential tumor suppressor roles for these genes (Fig. 3D).

### Recognition of MRG clusters

**Figure 4:**
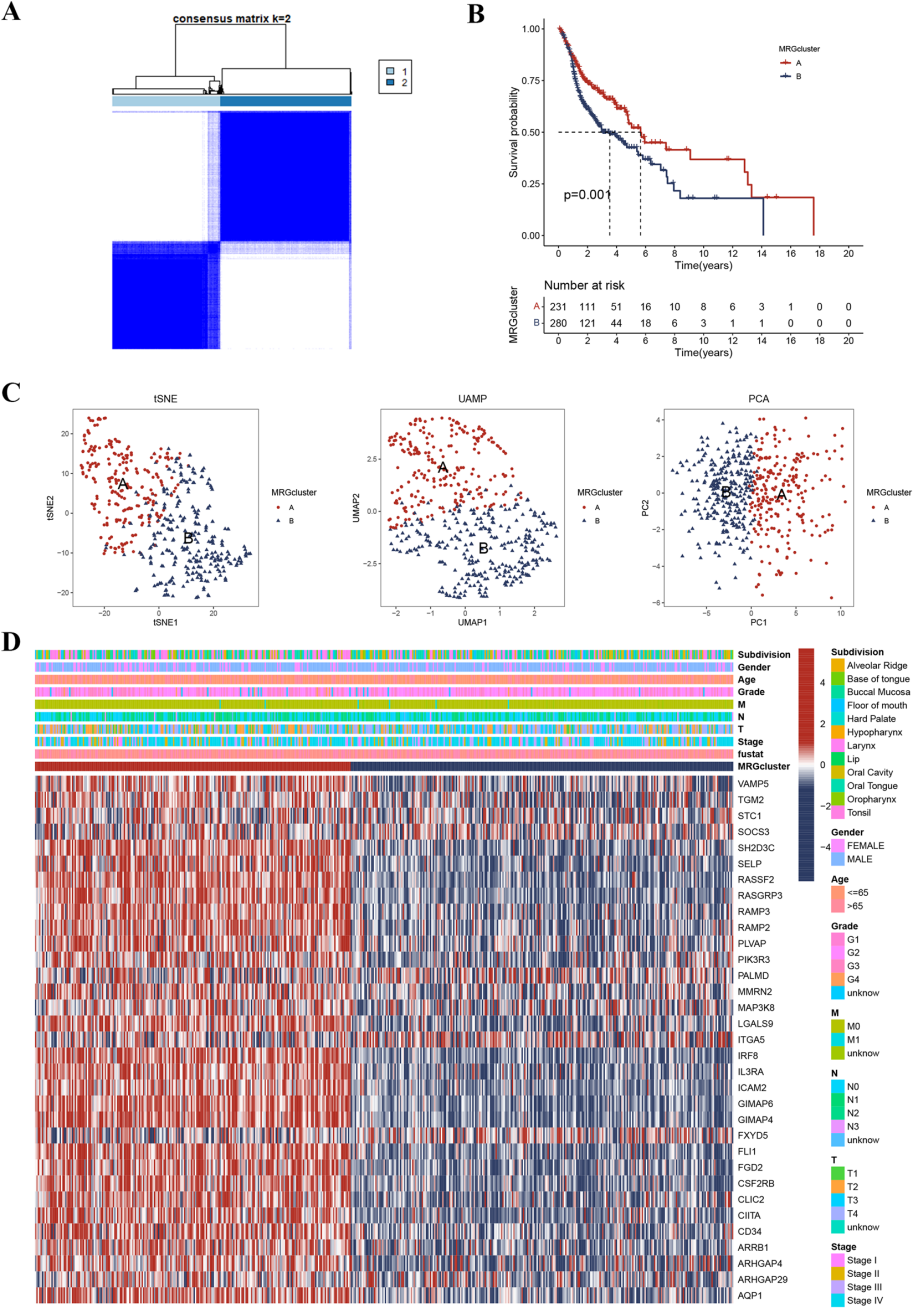
(**A**) Consensus matrix at optimal k = 2. (**B**) Patients in MRG cluster A had a significantly better survival probability. (**C**) The reliability of this classification was further corroborated by results obtained from PCA, tSNE, and UMAP methods. (**D**) Significant differences existed among the two groups in terms of clinical characteristics.

This study employed the consensus clustering approach to divide patients into distinct clusters from their MRG expression profiles. The optimal K value was selected based on various criteria. Based on these criteria, K = 2 was determined to be the most suitable choice (Figs. 4A and S1). Kaplan–Meier analysis indicated that MRG group A patients had a significantly better survival probability (Fig. 4B). The reliability of this classification was further corroborated by results obtained from PCA, tSNE, and UMAP methods, which revealed significant differences among different groups (Fig. 4C). Further analysis indicated significant differences among different groups in clinical and pathological characteristics (Fig. 4D).

### Exploration of immunoscape and enrichment analysis

**Figure 5:**
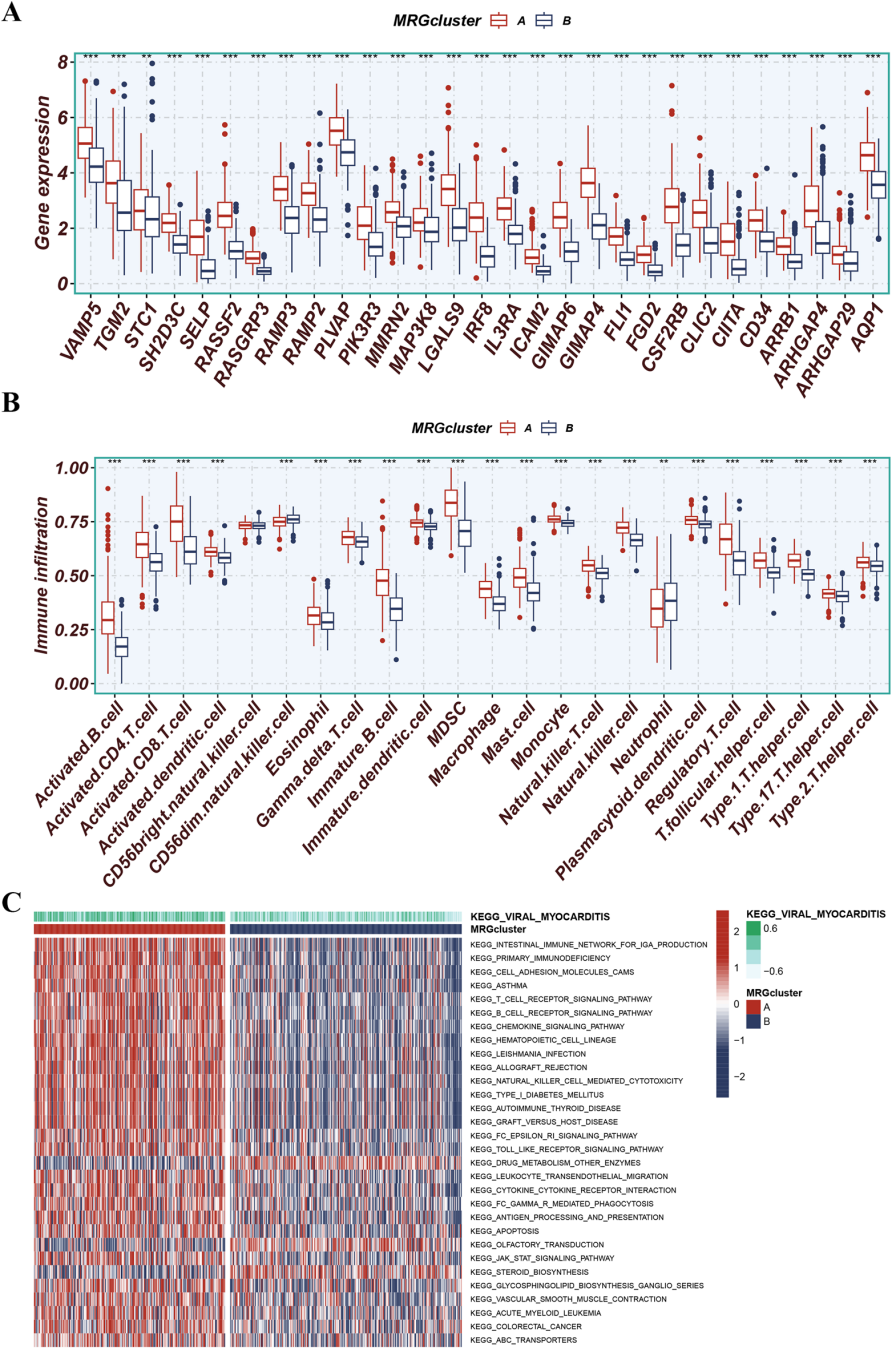
(**A**) MRG cluster A displayed significantly higher expression levels of MRGs. (**B**) The level of immune cell infiltration was significantly higher in MRG cluster A. (**C**) MRG cluster A was significantly enriched in pathways associated with intestinal immune network for IGA production, primary immunodeficiency, cell adhesion molecules CAMs, asthma, and T cell receptor signaling pathway. MRG cluster B was significantly enriched in pathways associated with drug metabolism other enzymes, olfactory transduction, steroid biosynthesis, retinol metabolism, and fructose and mannose metabolism.

To obtain the biological and immunological roles of the identified MRG clusters, the expression levels of MRGs in each cluster were analyzed. MRG cluster A displayed significantly higher expression of MRGs (Fig. 5A). The level of immune cell infiltration was significantly higher in MRG cluster A (Fig. 5B). In addition, the study employed the GSVA method to identify differential pathways that were enriched in each MRG cluster. MRG cluster A was significantly enriched in pathways associated with intestinal immune network for IGA production, primary immunodeficiency, cell adhesion molecules, asthma, and T cell receptor signaling pathway. On the other hand, MRG cluster B was significantly enriched in pathways associated with drug metabolism, other enzymes, olfactory transduction, steroid biosynthesis, retinol metabolism, and fructose and mannose metabolism (Fig. 5C).

### Construction and validation of MRGs signature

**Figure 6:**
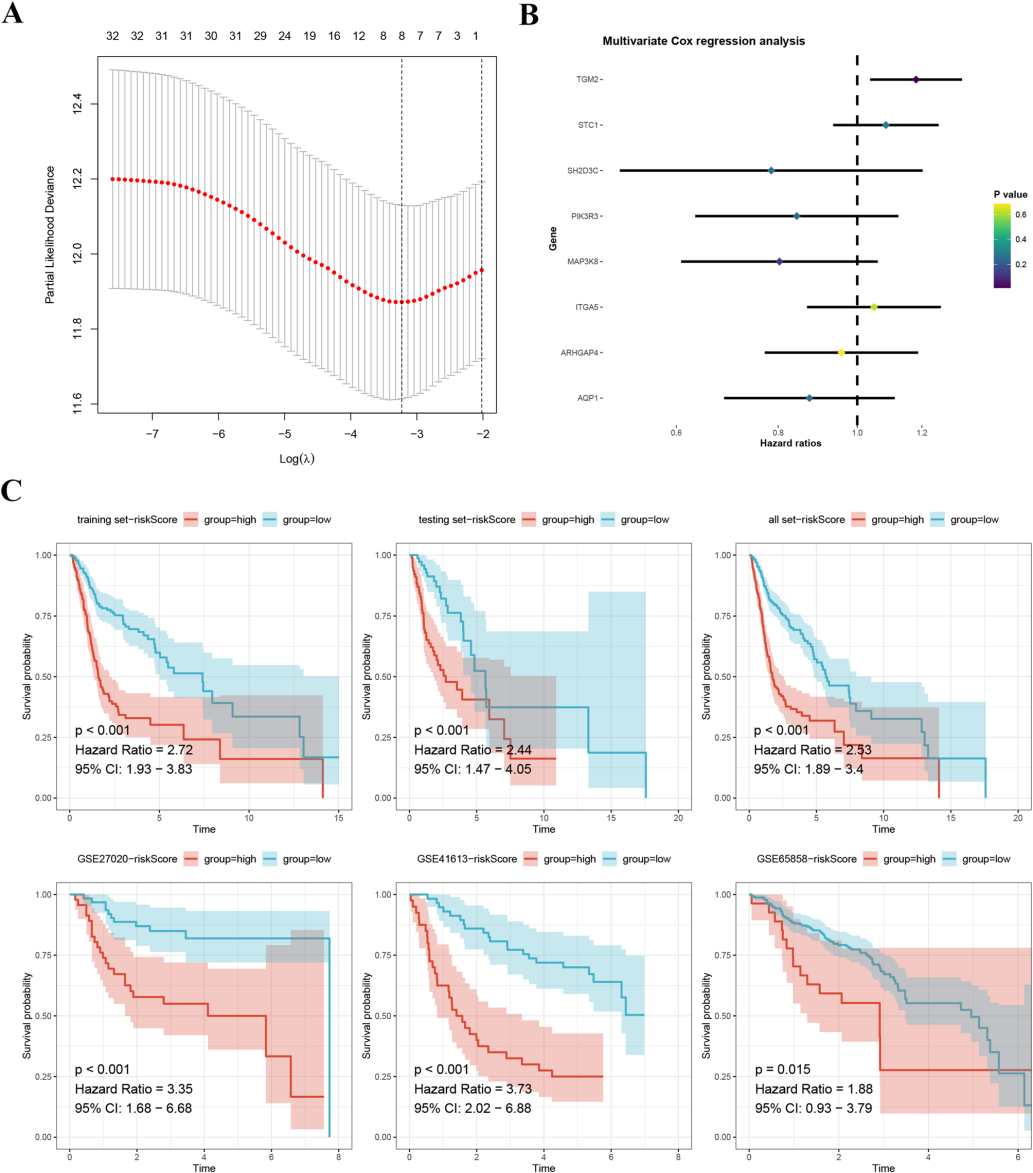
(**A**) 8 prognostic MRGs recognized by LASSO analysis. (**B**) A signature comprising 8 MRGs was developed using multivariate Cox analysis. (**C**) Patients classified as high-risk exhibited significantly shorter survival times.

**Figure 7:**
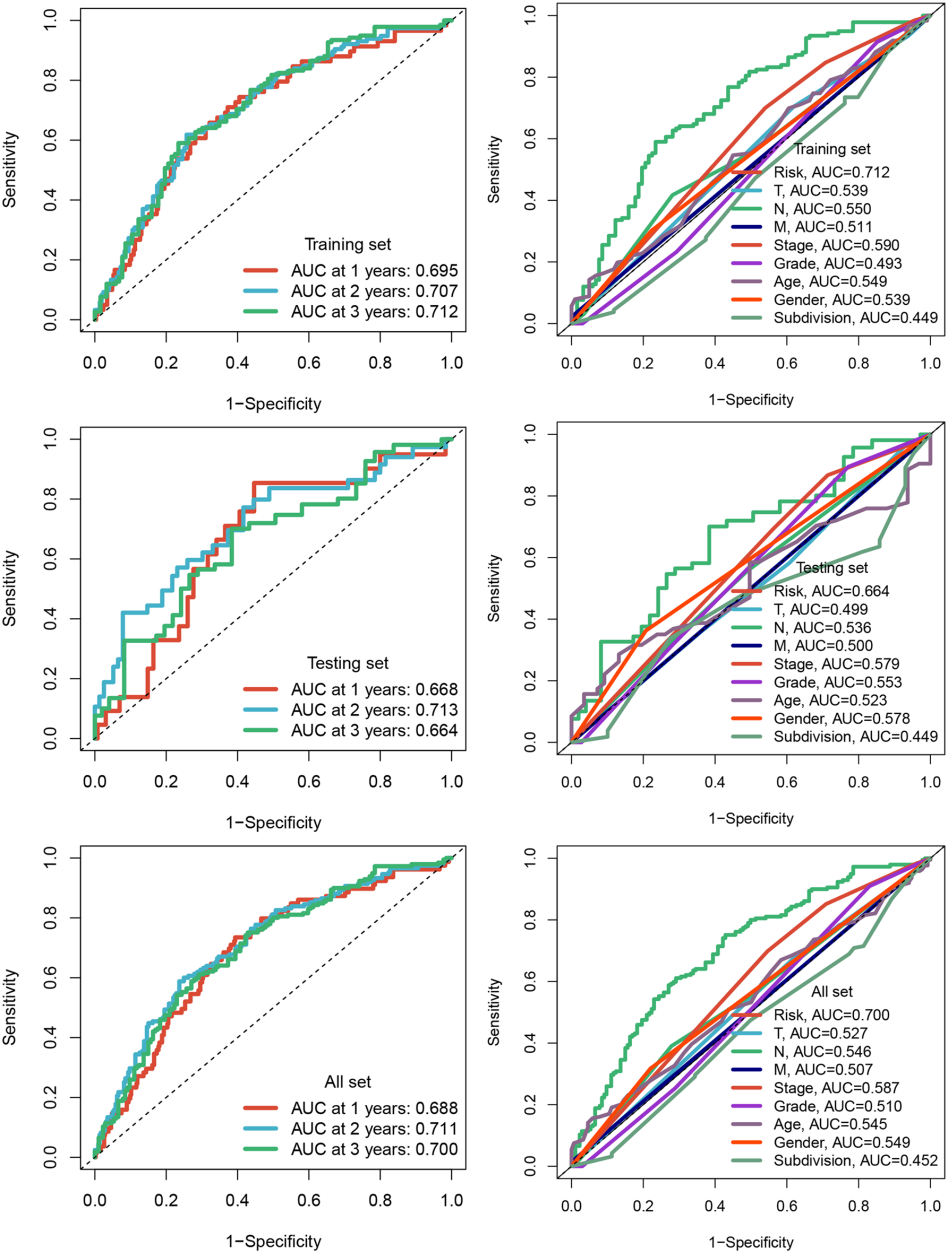
The MRG signature demonstrated the ability to predict survival rates in HNSCC patients. The AUC values obtained using the MRG signature were higher compared to those obtained using other clinical characteristics.

Three sets of HNSCC samples were utilized for analysis, including the training set (n = 358), the testing set (n = 153), and the entire set (n = 511), as well as three external validation sets, namely the GSE27020 set (n = 109), GSE41613 set (n = 97), and GSE65858 set (n = 270). The LASSO analysis was used to recognize a set of 33 prognostic MRGs, as depicted in Fig. 6A. Furthermore, multivariate Cox analysis was applied to construct a prognostic signature consisting of 8 MRGs (Fig. 6B). The tSNE and bubble plots indicated the distribution of expression levels of genes in the model, M1 macrophage markers and M2 macrophage markers (Fig S2). The high-risk patients exhibited shorter survival times (Fig. 6C). This finding was further validated using the external validation sets, wherein the high-risk group consistently showed shorter survival times, confirming the robustness of the MRG signature (Fig. 6C). Additionally, the newly established MRG signature demonstrated the ability to predict 1-, 2-, and 3-year survival rates in HNSCC, with AUC values of 0.688, 0.711, and 0.700 (Fig. 7). Importantly, the AUC values obtained using the MRG signature were higher compared to those obtained using other clinical characteristics, reinforcing the reliability and predictive power of the model (Fig. 7).

**Figure 8:**
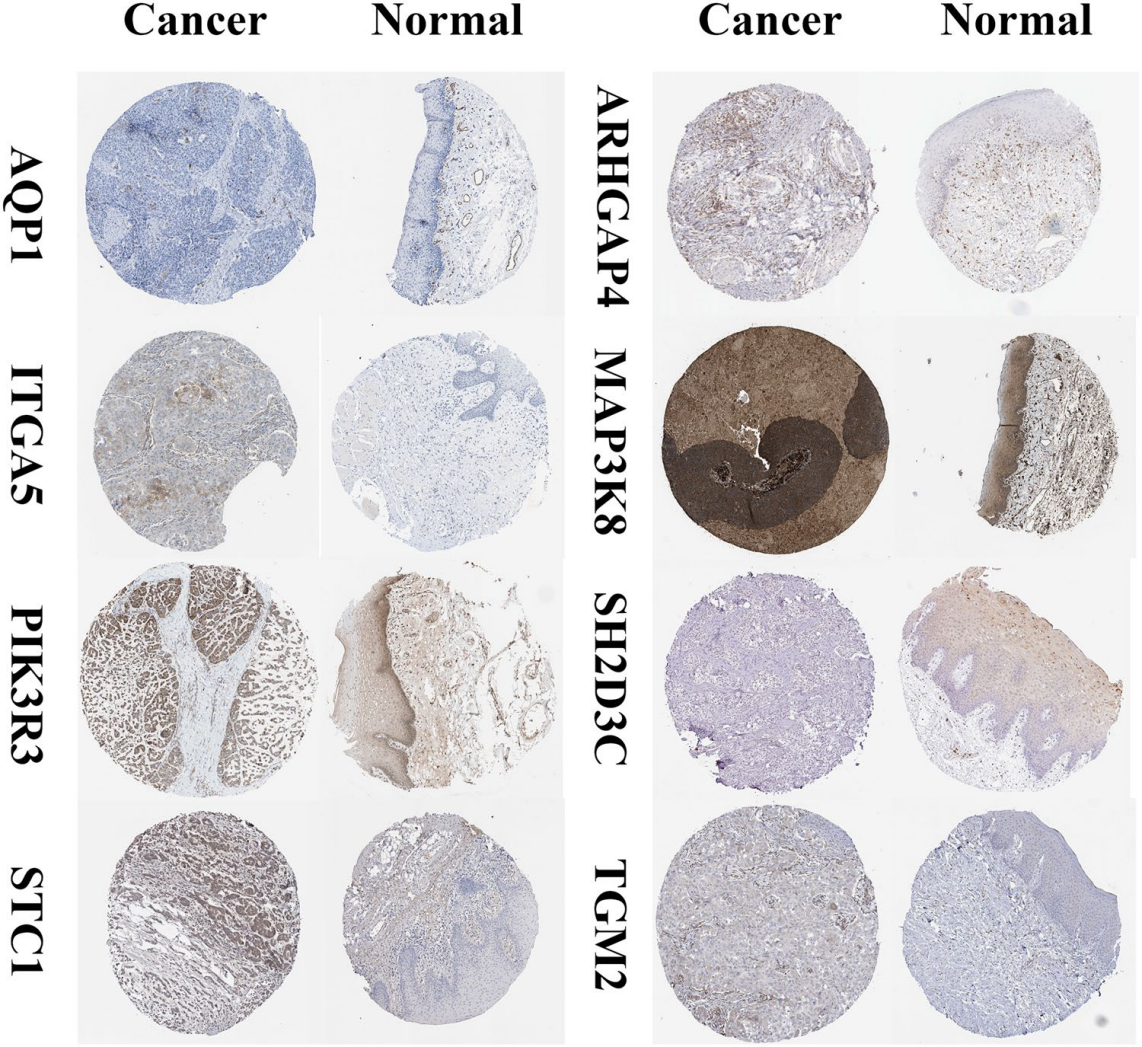
Through analysis of the HPA database, we identified significantly higher expression levels of specific proteins, including AQP1, ITGA5, MAP3K8, PIK3R3, STC1, and TGM2, in HNSCC tumor tissues. Conversely, SH2D3C exhibited significantly lower expression levels in HNSCC tumor tissues, while ARHGAP4 did not show differential expression.

**Figure 9:**
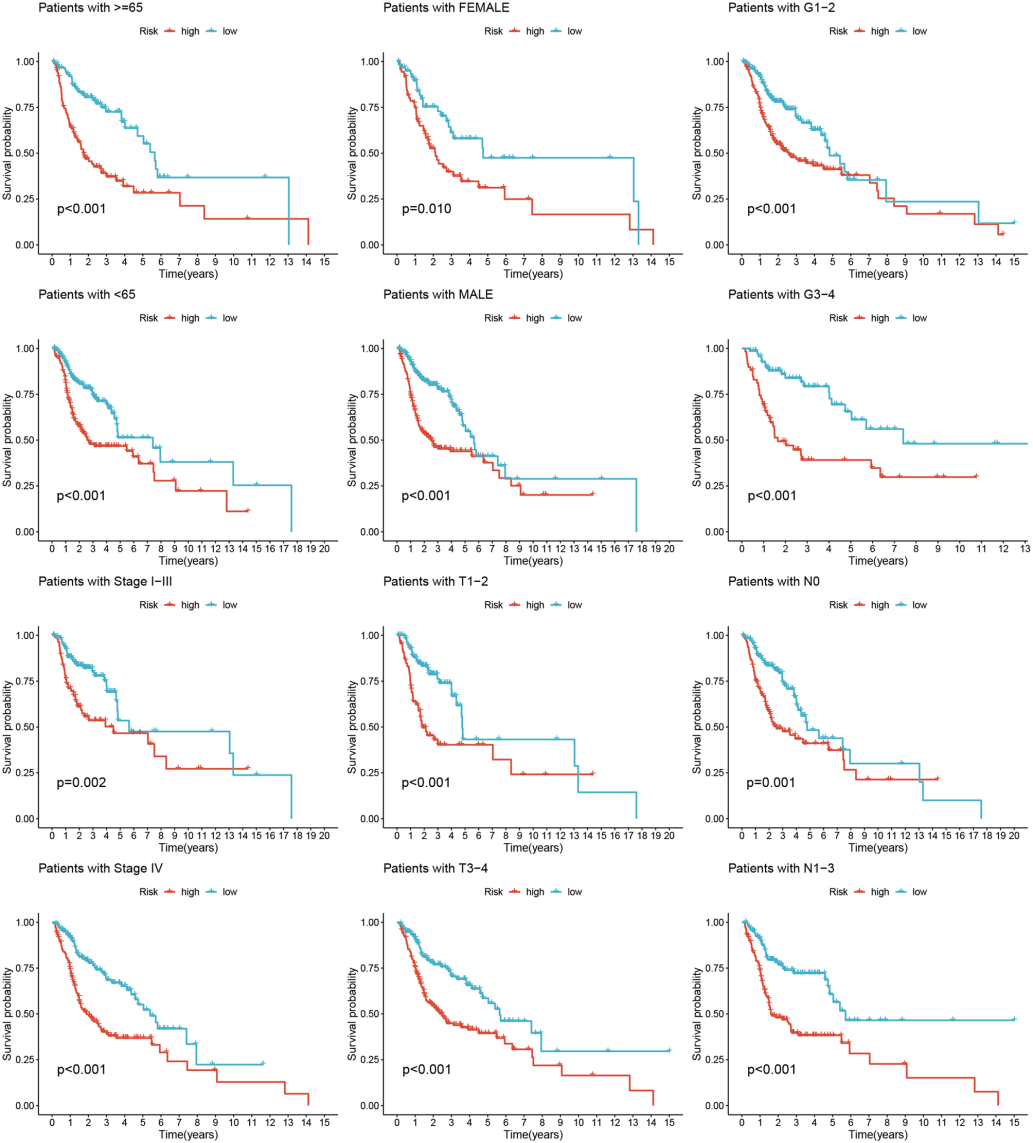
The low-risk group patients indicated higher survival rates across clinical subsets.

**Figure 10:**
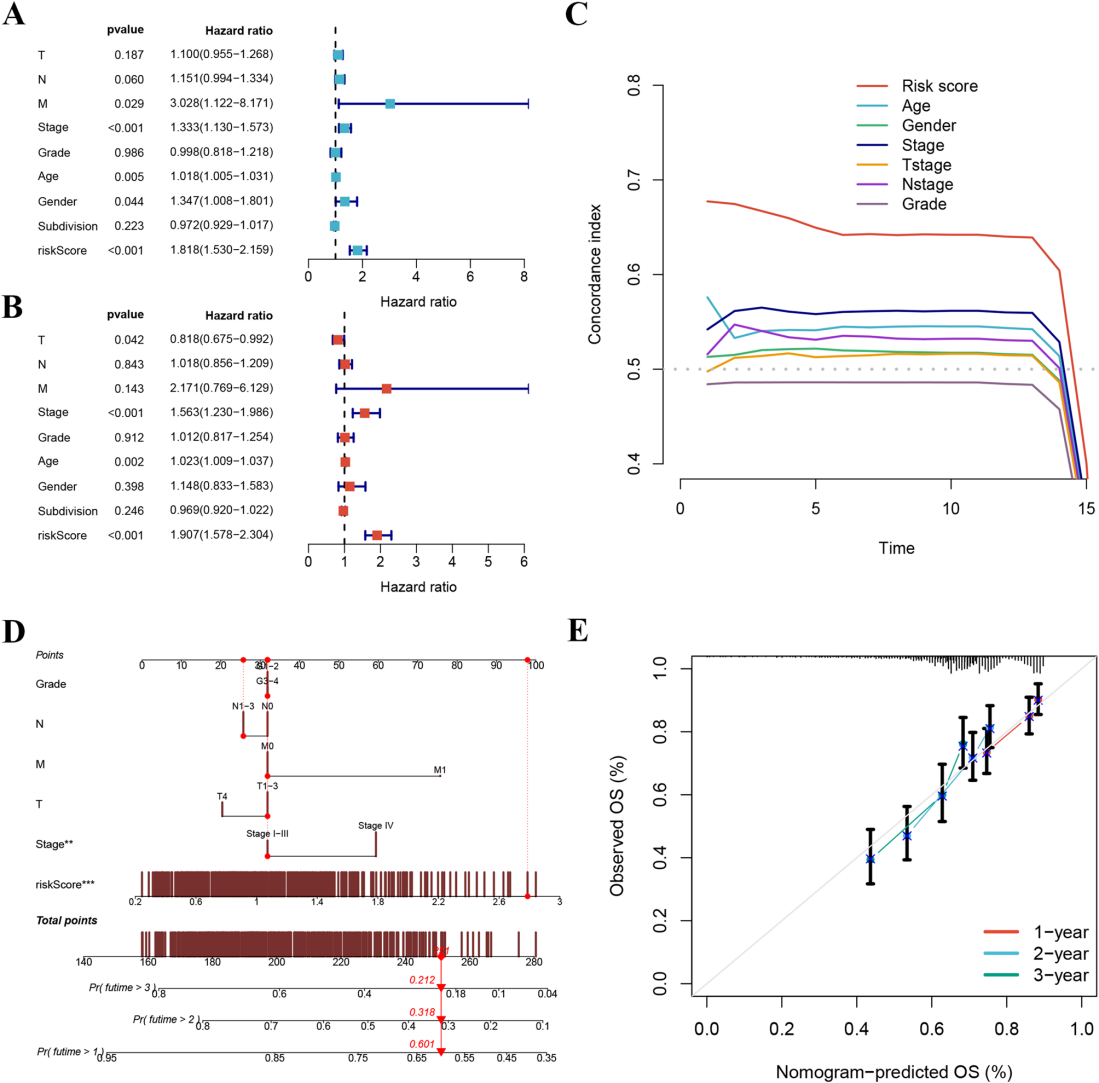
(**A** and **B**) The risk score was an independent prognostic factor. (**C**) C-index confirmed the superior prognostic performance of the signature compared to clinical features. (**D** and **E**) The nomogram demonstrates reliable and sensitive predictions for survival outcomes.

Through analysis of the HPA database, we recognized remarkably higher expression levels of specific proteins, including AQP1, ITGA5, MAP3K8, PIK3R3, STC1, and TGM2, in HNSCC tumor tissues. Conversely, SH2D3C exhibited remarkably lower expression levels in HNSCC tissues, whereas ARHGAP4 did not show differential expression (Fig. 8). Furthermore, the applicability of the MRG signature to different patient populations was assessed. The lower-risk group patients consistently demonstrated higher survival rates across various clinical subsets, which highlights the usefulness and generalizability of the model in diverse patient populations (Fig. 9). Both univariate and multivariate Cox analyses demonstrated that the risk score was an independent prognostic factor for HNSCC (Fig. 10A and B). Moreover, the C-index further confirmed the superior prognostic performance of the signature compared to clinical features (Fig. 10C). In order to be able to accurately predict the survival of HNSCC, the research developed a nomogram integrating the MRG signature and clinical features (Fig. 10D and E). The nomogram can be used as a useful instrument for clinical decision-making and personalized medicine, providing clinicians with a quantitative and individualized prediction model for HNSCC patient survival.

### Enrichment and mutation analysis

**Figure 11:**
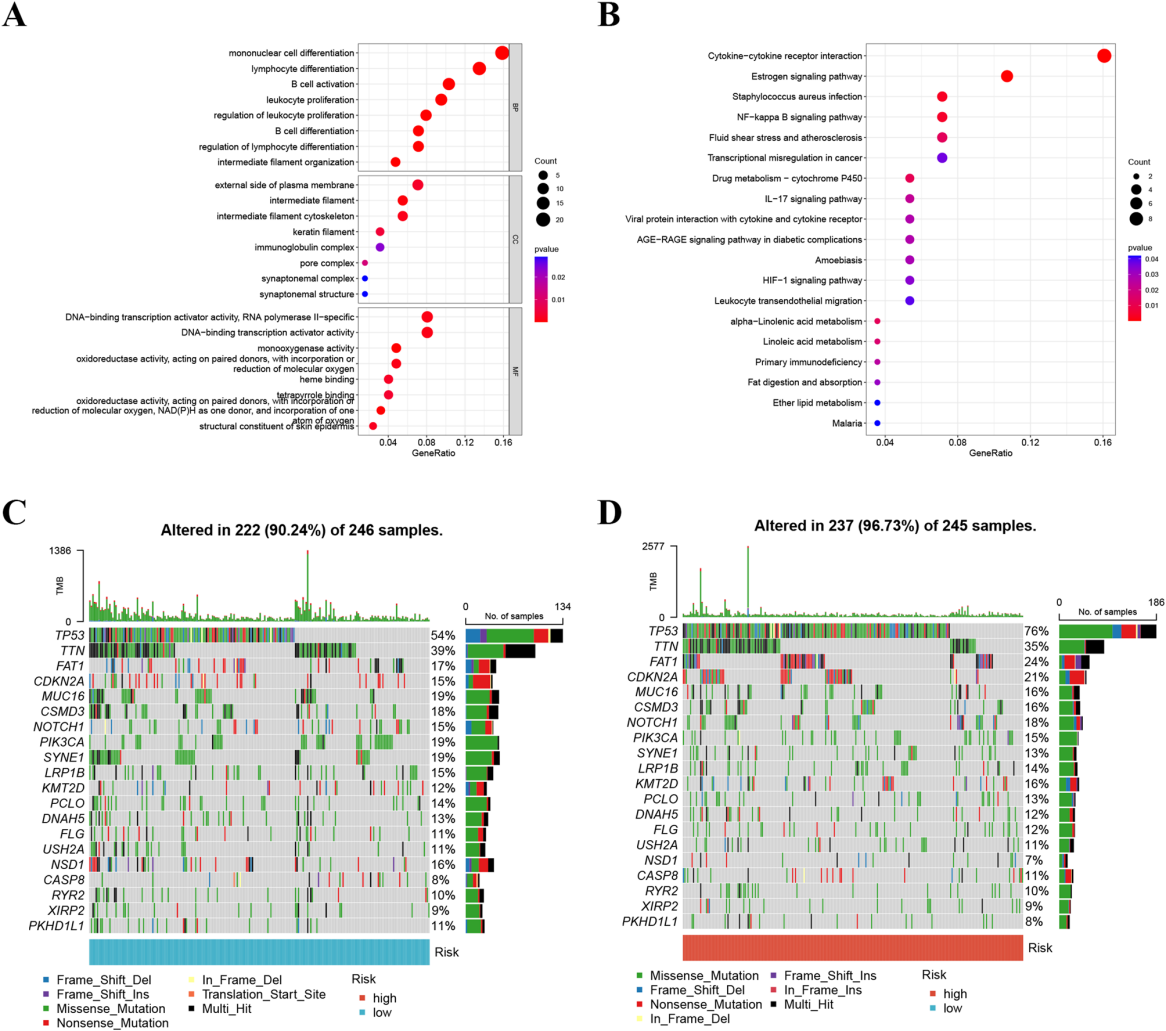
(**A** and **B**) The GO and KEGG analyses for 132 DEGs. (**C** and **D**) The top 10 genes with mutations were identified, and the frequency of mutations was higher in the high-risk group.

132 DEGs were recognized among various groups (|logFC >  = 1.5| and FDR < 0.05) (Table S2). BP terms showed enrichment for regulation of mononuclear cell differentiation, lymphocyte differentiation, and B cell activation. CC terms were associated with intermediate filament, intermediate filament cytoskeleton, and the external side of the plasma membrane. MF terms were associated with monooxygenase activity, oxidoreductase activity, and DNA-binding transcription activator activity (Fig. 11A and Table S3). Additionally, KEGG analysis showed that DEGs were highly enriched in pathways including cytokine-cytokine receptor interaction, estrogen signaling pathway, Staphylococcus aureus infection, NF-kappa B signaling pathway, and drug metabolism (Fig. 11B and Table S4). Moreover, the top 10 genes with mutations were identified, and the frequency of mutations was higher in the high-risk group Fig. 11C and D.

### Exploration of immunization status

**Figure 12:**
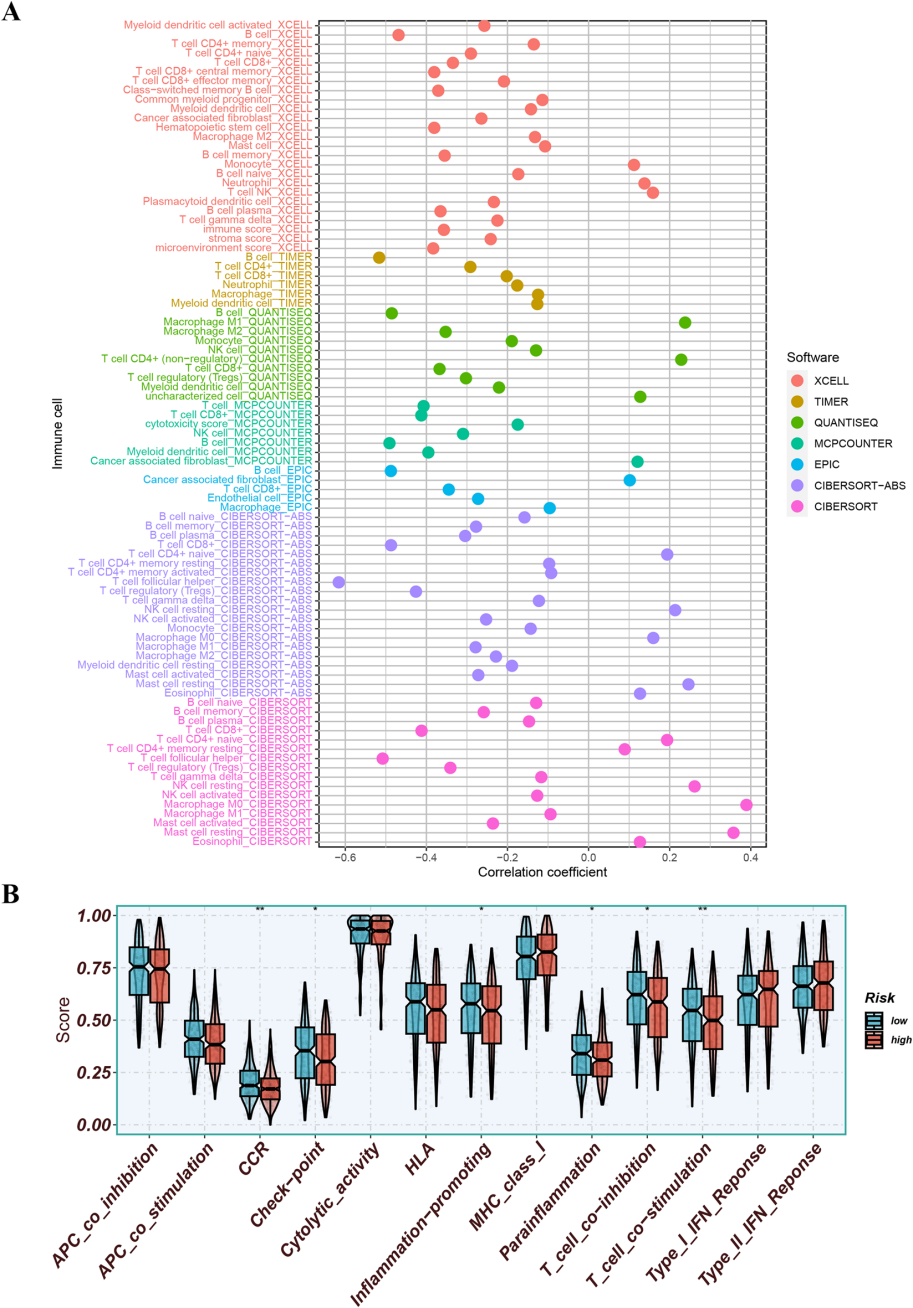
(**A**) Differences in immune cell subsets. (**B**) Differences in immune-related functions.

**Figure 13:**
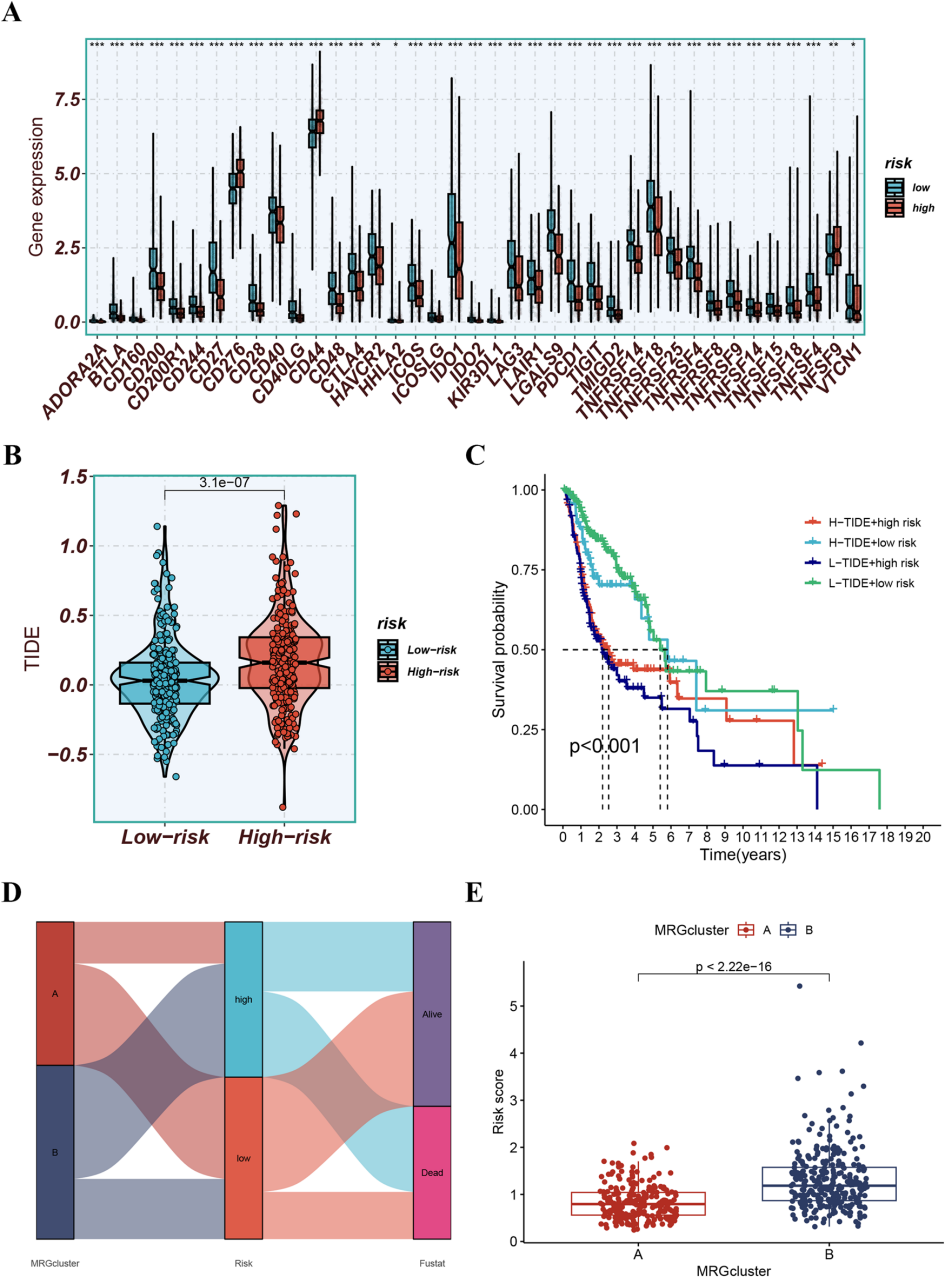
(**A**) The expression levels of various ICGs, including CTLA-4, TIGIT, LAG3, and IDO1, were significantly different between the risk groups. (**B**) Patients classified into the low-risk group demonstrated lower TIDE scores, indicating a possibility of higher responsiveness to immunotherapy. (**C**) Combining the TIDE scores provided a better prediction of patient prognosis. (**D** and **E**) The Sankey plot was informative in revealing the links between MRG clusters, risk groups, and survival status. It showed that exposure to MRG cluster B was associated with higher risk scores.

Significant differences in the levels of immune cell subsets including NK cells, B cells, myeloid dendritic cells, cancer-associated fibroblasts, macrophages, mast cells, CD4 + T cells, CD8 + T cells, and regulatory T cells were observed in different groups of immune cell subsets (Fig. 12A). Moreover, differences in immune-related functions such as inflammation-promoting, parainflammation, CCR, checkpoint, T cell co-inhibition, and T cell co-stimulation were observed (Fig. 12B). Furthermore, this study found that the expression levels of several ICGs, including CTLA-4, TIGIT, LAG3, and IDO1, were significantly different between the risk groups (Fig. 13A). Additionally, patients classified into the low-risk group demonstrated lower TIDE scores, indicating a possibility of higher responsiveness to immunotherapy (Fig. 13B). Combining the TIDE scores provided a better prediction of patient prognosis (Fig. 13C). The Sankey plot was informative in revealing the links between MRG clusters, risk groups, and survival status. It showed that exposure to MRG cluster B was related to higher risk scores Fig. 13D and E.

### Identification of drugs and correlation with malignant features

**Figure 14:**
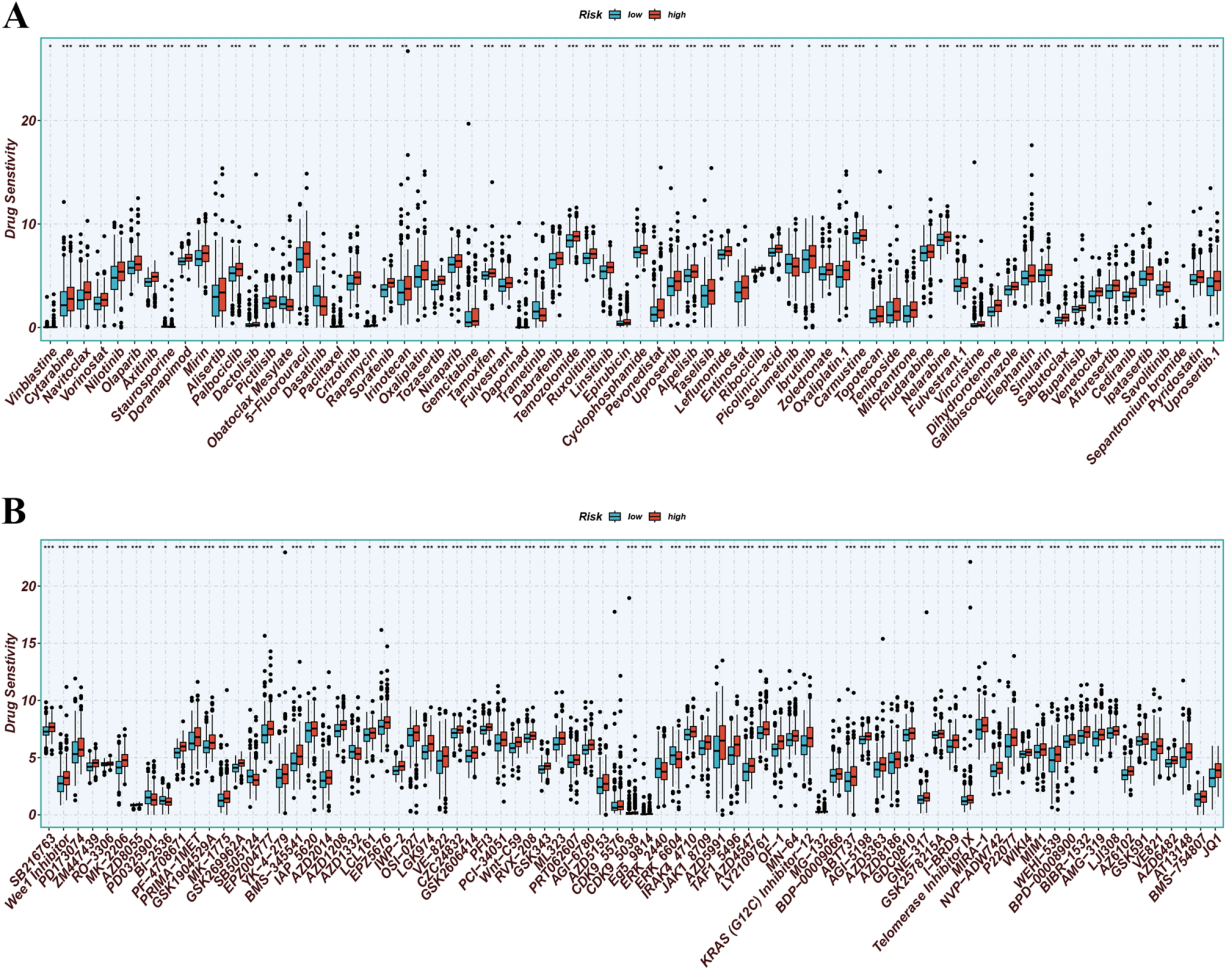
(**A** and **B**) A search for chemotherapeutic medications and targeted agents was conducted, and customized therapy regimens were developed based on patient subgroups (*p* < 0.05).

**Figure 15:**
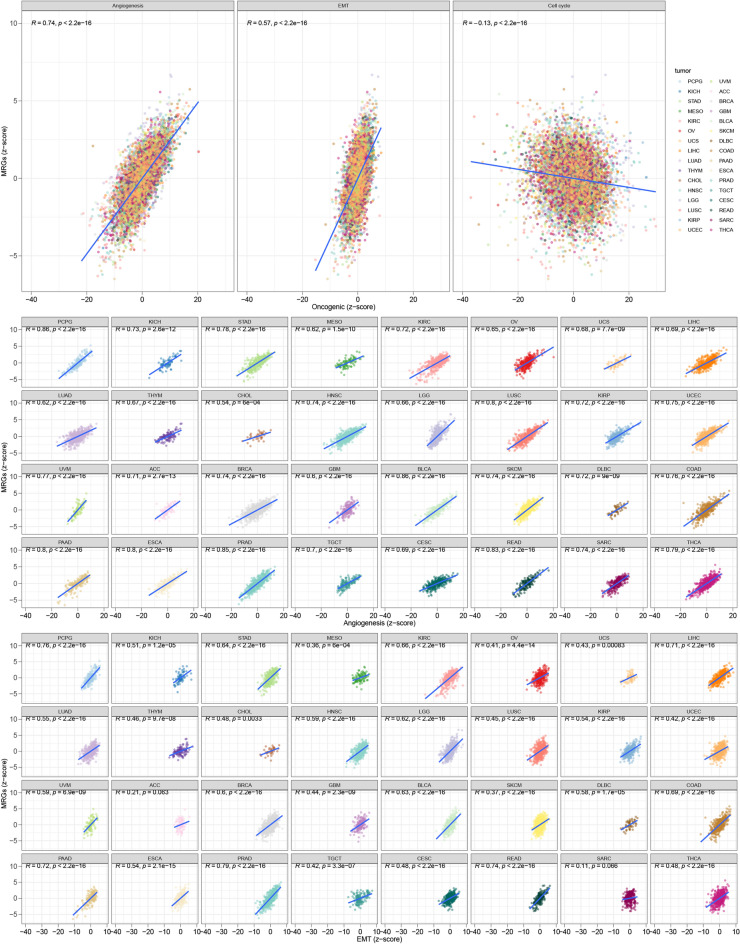
Significant correlations between the MRG z-score and angiogenesis z-score (R = 0.74, *p* < 0.001), EMT z-score (R = 0.57, *p* < 0.001), and cell cycle z-score (R = -0.13, *p* < 0.001).

70 chemotherapeutic drugs and 81 targeted therapeutic drugs were found, including 5-Fluorouracil, Temozolomide, Carmustine, and EPZ5676, etc. (*p* < 0.05; Fig. 14A and B). Correlation analysis indicated significant correlations between the MRG z-score and angiogenesis z-score (R = 0.74, *p* < 0.001), EMT z-score (R = 0.57, *p* < 0.001), and cell cycle z-score (R = − 0.13, *p* < 0.001) across the TCGA pan-cancer cohort (Fig. 15).

## Discussion

In the early stage of HNSCC, surgical treatment can often achieve good results, but in the middle and late stages of HNSCC, even if comprehensive treatment such as radiotherapy combined with systemic therapy is used after surgery, the 5-year survival rate is not optimistic, and the incidence of recurrent tumors is as high as 60%^3,55–57^. Immune checkpoint inhibitors have demonstrated good anti-tumor ability by the marker KCSG HN18-12 in patients with HNSCC who have relapsed and advanced metastases after chemotherapy^58^. Li et al.^20^ also comprehensively elaborated on the key role of TAM in regulating the progression of head and neck tumors, revealing its immunosuppressive effect and potential mechanism of action in tumor tissues. With the increasing research on tumor immunotherapy, treatments including immune checkpoint inhibitors have also brought hope to intermediate and advanced HNSCC patients^59–61^.

TAMs are multifunctional tumor-associated immune cells, which often exhibit a variety of different phenotypes according to different tumor types and different TMEs, and the most common phenotypes are M1, M2, etc.^62^. M1 macrophages are considered to be "tumor killer macrophages", secreting pro-inflammatory cytokines, including IL-12, TNF-α, etc., mainly anti-tumor and promote immunity. It has been demonstrated that M1 exosome lncRNA HOTTIP can up-regulate the TLR5/NF-κB pathway by competitive spongy miR-19a-3p and miR-19b-3p, and M1 exosomes and HOTTIP can induce M1 polarization in circulating monocytes, thereby inhibiting the progression of HNSCC^63^. Gene knockout assays have shown that RGS12 can inhibit the progression of oral squamous cell carcinoma by controlling the polarization of M1 in TAMs by controlling ciliated MYCBP2/KIF2A signaling^64^. The M2 type is the opposite, it secretes anti-inflammatory cytokines and is often thought to be an expression subtype that promotes tumor development.^62,65–67^. Gao et al. used immunohistochemistry and immunofluorescence staining to detect TAM biomarkers and EMT-related proteins, and found that the expression of EMT-related proteins was positively correlated with M2 macrophage biomarkers in HNSCC tissues, revealing that the M2 type of TAMs may induce the EMT process in cancer cells by activating the EGFR/ERK1/2 signaling pathway in HNSCC and then promote tumors^68^. Through meta-analysis, Ayan et al. showed that the higher density of total TAMs and M2-like subtype TAMs in the TME was associated with T stage progression, nodule positivity, vascular invasion and lymphatic invasion^69^. TAMs are highly related to HNSCC and can help immunotherapy with HNSCC, so MRGs deserve to be explored in depth^70–72^. Therefore, the importance of treating HNSCC accurately and identifying more models helps to control HNSCC, construct a reasonable prognostic risk model, evaluate the correlation with tumor malignant characteristics, explore the immune differences of tumor tissues, and screen out more and more reasonable anti-tumor drugs.

In the study, we first searched for RNA-seq and clinical data related to HNSCC in the open databases GEO-GSE103322, TCGA-HNSCC, GEO-GSE27020, GEO-GSE41613, and GEO-GSE65858. After performing data quality control, we found that 33 MRGs (such as LI1, RASSF2 and IL3RA, etc.) showed the potential for disease prognosis prediction and were significantly related to survival, of which 18 genes have potential carcinogenic effects, and 15 other genes have potential cancer suppression. For these genes, some researchers have found that ARRB1 can promote the activation of the TAK1/MAPK pathway to promote the progression of gallbladder cancer, and ARHGAP4 can be targeted by miR-939-5p, thereby promoting the invasion and metastasis of pancreatic cancer^73,74^. Experiments have shown that FXYD5 can promote metastasis of mouse breast cancer tissues by regulating the β-Na + -K + -ATPase subunit^75^. Similarly, in another type of gene, the gene RASSF2 was shown to be an oncogene for Ewing sarcoma, and high GIMAP4 expression could facilitate the TME of immune cells to detect tumor cells and inhibit the development of lung adenocarcinoma cells^76,77^. There are many similar scientific studies mentioned above, and the effects of these genes on tumors in the above studies are consistent and trustworthy with the conclusions of this study. The consensus clustering method divided patients into different clusters, and the survival probability analysis showed that the survival rate of group A patients was much higher. We found that they had different features by analyzing differences in clinical features among different subtypes, suggesting that this might be a useful prognostic marker for patients with HNSCC, which will likely guide our clinical work. Gene expression analysis showed that cluster A was more closely correlated with MRG, and GSEA analysis found that the immune cell infiltration level of cluster A was significantly increased. GSVA analysis found that cluster A was significantly enriched in pathways related to IGA production and primary immunodeficiency, while cluster B was more enriched in drug metabolism and olfactory transduction of other enzymes. In the face of different enrichment states, this will be able to guide us to differentiated treatment of patients.

In this study, 3 sets of HNSCC samples were used for analysis, 33 prognostic MRGs were identified by LASSO analysis, and a prognostic model consisting of 8 MRGs (TGM2, STC1, SH2D3C, PIK3R3, MAP3K8, ITGA5, ARHGAP4 and AQP1) was constructed by multivariate Cox analysis. The survival prognosis was notably worse in the high-risk group, and this finding was further validated by the external validation set. The newly established MRG model can better forecast the survival rates of HNSCC, and its higher AUC value enhances the reliability of the model, which will guide whether clinical treatment is aggressive or palliative. In this study, AQP1, ITGA5, MAP3K8, PIK3R3, STC1 and TGM2 were significantly overexpressed in HNSCC tumor tissues, while SH2D3C expression was significantly reduced, and there was no difference in ARHGAP4 expression. This is similar to the results of "Guo et al. found that AQP1 can promote local invasion of breast cancer, Xu et al. found that ITGA5 can promote angiogenesis in cervical cancer, and Lee et al. found that MAP3K8 overexpression can induce squamous cell carcinogenesis in the salivary glands of mice"^78–80^. Studies have shown that STC1 is not only one of MRGs, but also a glycolysis-related genes in HNSCC, which is prominently expressed in the glycolytic activities of tumor tissues and can also predict the prognosis of HNSCC from the direction of glycolysis, which is similar to the conclusion of this study^81^. In this study, we also integrated MRG features and clinical features to develop a nomogram, which will provide an excellent tool for clinical and scientific work.

At the same time, we identified 132 DEGs between different groups, and GO analysis and KEGG analysis further indicated the different roles of DEGs. In addition, we analyzed mutations in the first 10 typical genes, whose mutations might facilitate the progression of HNSCC. Among them, TP53 and other genes are hot genes in the research of HNSCC, which has great immunotherapy prospects^82–85^. Studies have confirmed that FAT1 mutations are highly abundant in cisplatin responders in HNSCC patients, and the gene has been found to have potential targetable changes in 15% of HNSCC patients, which is great news for targeted immunotherapy^86^. The immune landscape study found significant differences in multiple immune cell subsets and in several ICG groups, and the low-risk group showed a lower TIDE score, suggesting a possible higher response to immunotherapy. Therefore, pooled TIDE scores provide a better predictor of patient outcomes.

Through the search for various chemotherapy drugs and innovative targeted drugs, this may lead to the generation of personalized treatment regimens for patients belonging to different subgroups. The more prominent drugs are 5-Fluorouracil, Temozolomide, Carmustine, and EPZ5676, etc. At the end of the study, using the z-scoring algorithm, we found significant associations with angiogenesis, EMT, and cell cycle in the TCGA cancer cohort.

Even so, our research had certain limitations. Firstly, the research is based on an analysis of public databases, which is a retrospective study. Even if scientific verification methods are used in the study, the absolute accuracy and applicability of the relevant conclusions cannot be guaranteed, and a large amount of experimental data and clinical data are still required to verify the relevant models and conclusions.

## Conclusion

We constructed a prognostic signature consisting of 8 MRGs (TGM2, STC1, SH2D3C, PIK3R3, MAP3K8, ITGA5, ARHGAP4 and AQP1) by multivariate Cox analysis. The low-risk group of patients may have a higher response to immunotherapy. In terms of drug selection, the more prominent drugs are 5-Fluorouracil, Temozolomide and so on. Angiogenesis, EMT, and cell cycle are malignant features associated with HNSCC. This study opens up new prospects for the prognosis prediction and clinical treatment strategy of HNSCC.

### Supplementary Information

Supplementary Figure S1.

Supplementary Figure S2.

Supplementary Legends.

Supplementary Tables.

## Data Availability

The datasets analyzed during the current study are available from the corresponding author on reasonable request.
