# Supplementary figures and images for: Characterization of macrophages in head and neck squamous cell carcinoma and development of MRG-based risk signature
Source: Sci Rep. 2024 Apr 30;14:9914. doi: 10.1038/s41598-024-60516-6 (PMC11061135; doi:10.1038/s41598-024-60516-6)

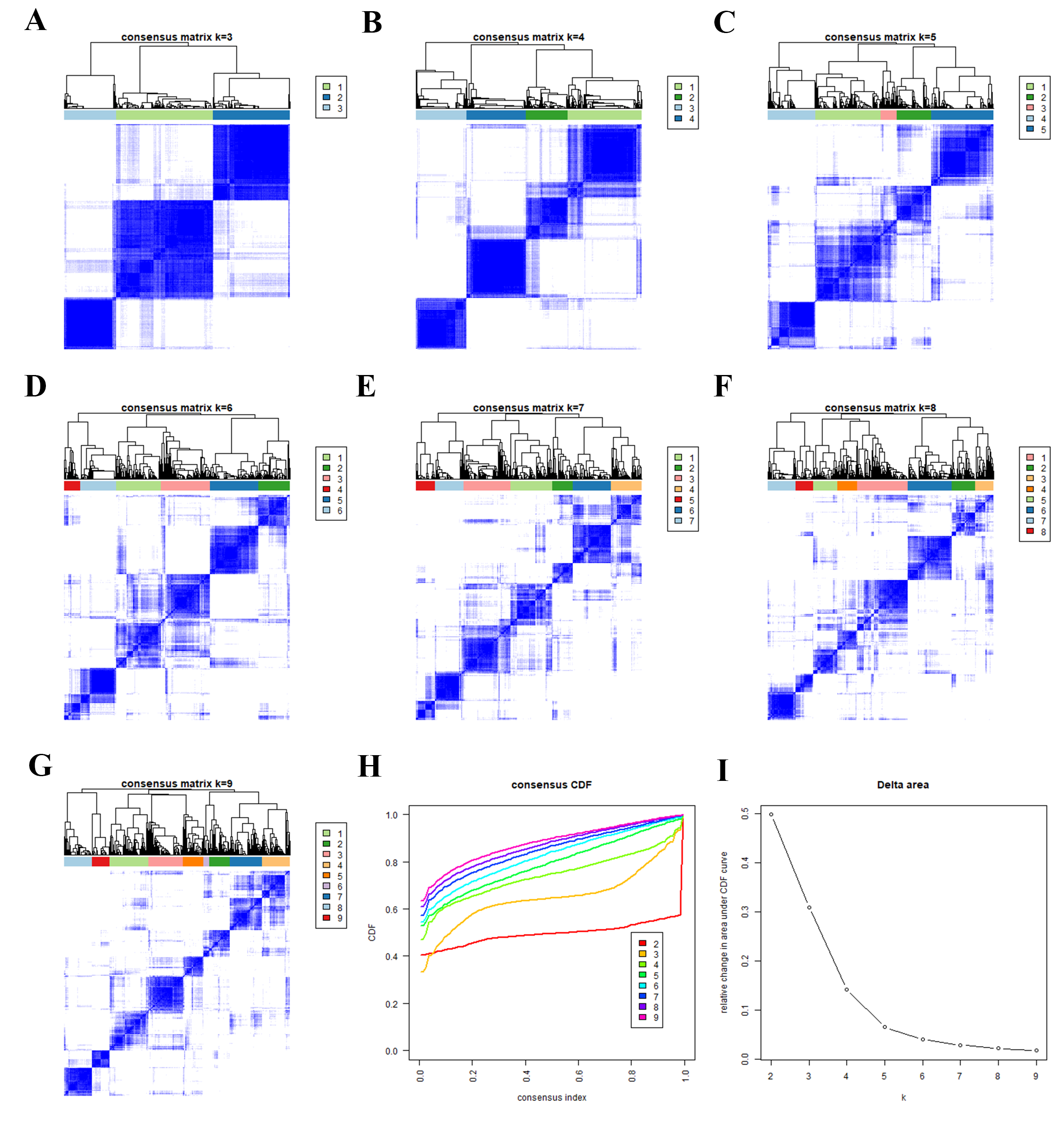

Supplement: Supplementary file 1 — Supplementary Figure S1. [file 41598_2024_60516_MOESM1_ESM.tif]

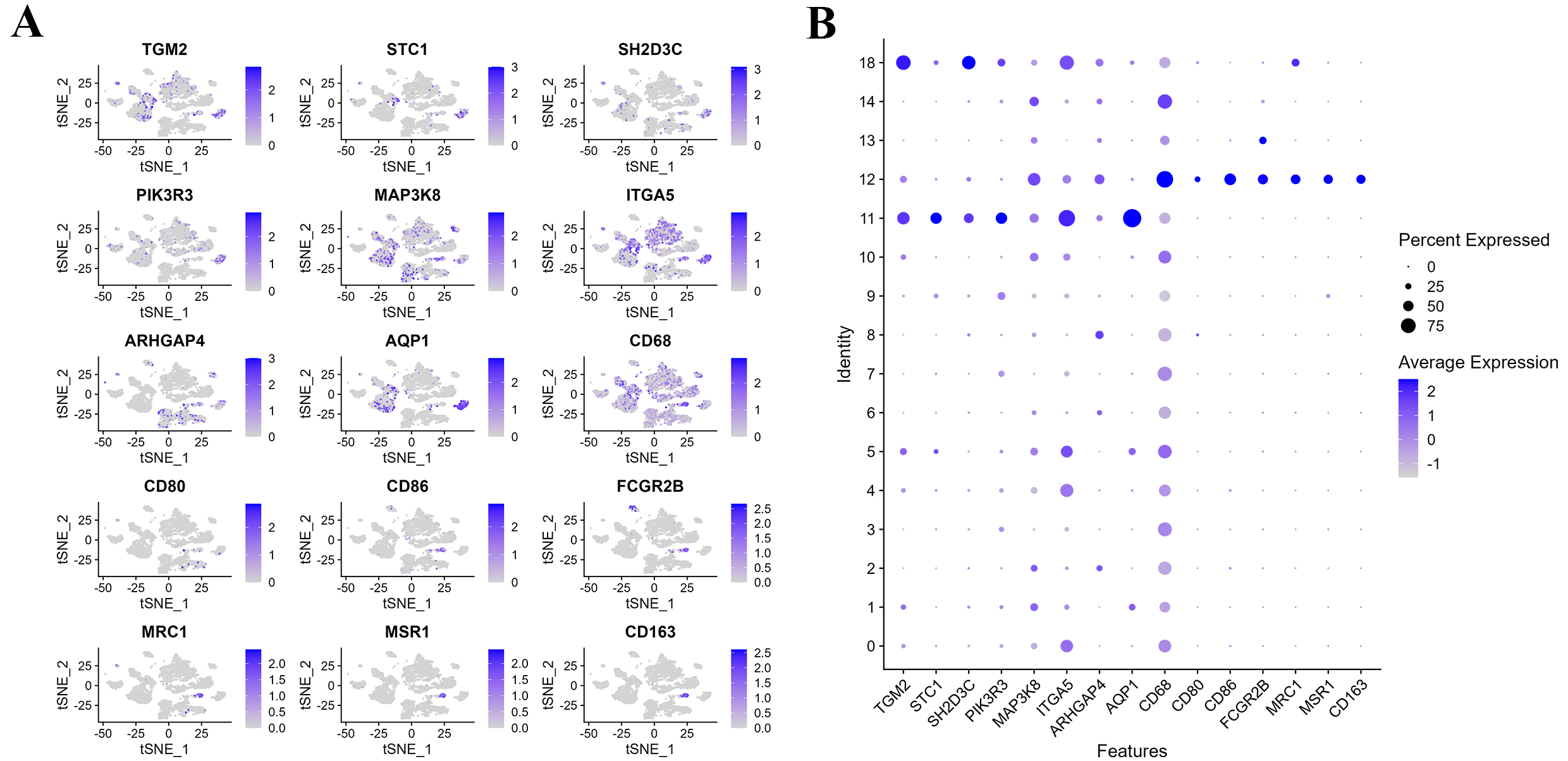

Supplement: Supplementary file 2 — Supplementary Figure S2. [file 41598_2024_60516_MOESM2_ESM.tif]
