# Supplementary material for: Characterization of macrophages in head and neck squamous cell carcinoma and development of MRG-based risk signature
Source: Sci Rep. 2024 Apr 30;14:9914. doi: 10.1038/s41598-024-60516-6 (PMC11061135; doi:10.1038/s41598-024-60516-6)
Supplement: Supplementary file 3 — Supplementary Legends. [file 41598_2024_60516_MOESM3_ESM.docx]

**Fig S1**. (A-G) Consensus matrices of the MRGs for k = 3-9. (H and I) The cumulative distribution function plot and the consensus clustering matrix's delta area.

**Fig S2**. (A-B) The tSNE and bubble plots indicated the distribution of expression levels of genes in the model, M1 macrophage markers and M2 macrophage markers..

**Tab S1.** The 216 significantly differentially expressed MRGs.

**Tab S2.** The 132 DEGs.

**Tab S3.** The GO analysis.

**Tab S4.** The KEGG analysis.
